# SerpentinaDB: a database of plant-derived molecules of *Rauvolfia serpentina*

**DOI:** 10.1186/s12906-015-0683-7

**Published:** 2015-08-04

**Authors:** Shivalika Pathania, Sai Mukund Ramakrishnan, Vinay Randhawa, Ganesh Bagler

**Affiliations:** Biotechnology Division, CSIR-Institute of Himalayan Bioresource Technology, Council of Scientific and Industrial Research, Palampur, Himachal Pradesh, India; Centre for Biologically Inspired Systems Science, Indian Institute of Technology Jodhpur, Jodhpur, India; Academy of Scientific & Innovative Research (AcSIR), New Delhi, India

**Keywords:** Plant-derived molecules, *Rauvolfia serpentina*, Database, Drug discovery, Virtual screening, ADMET

## Abstract

**Background:**

Plant-derived molecules (PDMs) are known to be a rich source of diverse scaffolds that could serve as a basis for rational drug design. Structured compilation of phytochemicals from traditional medicinal plants can facilitate prospection for novel PDMs and their analogs as therapeutic agents. *Rauvolfia serpentina* is an important medicinal plant, endemic to Himalayan mountain ranges of Indian subcontinent, reported to be of immense therapeutic value against various diseases.

**Description:**

We present SerpentinaDB, a structured compilation of 147 *R. serpentina* PDMs, inclusive of their plant part source, chemical classification, IUPAC, SMILES, physicochemical properties, and 3D chemical structures with associated references. It also provides refined search option for identification of analogs of natural molecules against ZINC database at user-defined cut-off.

**Conclusion:**

SerpentinaDB is an exhaustive resource of *R. serpentina* molecules facilitating prospection for therapeutic molecules from a medicinally important source of natural products. It also provides refined search option to explore the neighborhood of chemical space against ZINC database to identify analogs of natural molecules obtained as leads. In a previous study, we have demonstrated the utility of this resource by identifying novel aldose reductase inhibitors towards intervention of complications of diabetes.

**Electronic supplementary material:**

The online version of this article (doi:10.1186/s12906-015-0683-7) contains supplementary material, which is available to authorized users.

## Background

Plants have evolved to produce a diverse repertoire of secondary metabolites which have been used as a source of remedial agents [1, 2]. Medicinal plant extracts have been known for their efficacy against various diseases, and are classically used to discover drug-like molecules. Phytomedicines continue to play a central role in the health management systems in developing countries which include 65 % of Indian population. Recent World Health Organization (WHO) review estimates that almost 80 % of world’s population depends on traditional medicines [3]. These indicators have impelled WHO to incorporate phytomedicines in health care systems. PDMs have also been recognized to provide specific substructures or scaffolds that make them comparable to trade drugs and their potential utilization in combinatorial chemistry [4].

Therefore, there is ample scope for rationalizing the process of drug discovery by prospecting for plant-derived molecules (PDMs) with virtual screening approach. PDMs could be effectively used to systematically extract unique molecular scaffolds, which could further be chemically elaborated to generate novel leads and to screen molecules from drug-like libraries [5, 6]. Computational approaches, such as molecular docking, ligand-based virtual screening, and molecular dynamics (MD), have been widely used in modern drug discovery to explore drug-receptor interactions, and have been able to restrain the number of PDMs that confront experimental validation that ultimately reducing the cost of drug development [1, 7–9]. Thus, hypothesis driven implementation of such pharmacoinformatics pipeline hastens the rate of drug discovery of natural molecules and their simpler mimetics with better pharmacological properties.

*Rauvolfia serpentina* is an important medicinal plant endemic to the Himalayan mountain range of Indian subcontinent and South-East Asian countries [10]. Plant extracts of *R. serpentina* have been reported to be of therapeutic value against various diseases (Table 1) including hypertension, intestinal disorders, eye diseases, cuts, wounds, splenic diseases, uterine contraction, headache, and skin diseases [11]. Its extracts have also been reported with a broad range of therapeutic effects such as antioxidant, antiaging, antihypertensive, anticancerous, antimalarial, antiinflammatory, antifibrillar, anthelmintic, antiarrhythmic, anticholinergic, antidysentry, antidiarrhoeal, antihypotensive, anticontractile, antipyretic, antidiuretic, sympathomimetic, and antipsychotic [10–12]. Knowing the potential efficacy of *R. serpentina* PDMs and their derivatives, its phytochemical space could be effectively explored for systematical extraction of unique molecular scaffolds and their derivatives [6]. This strategy has been followed to identify ‘2 PDM leads’ and their 16 structural analogs as potent aldose reductase inhibitors (Additional file 1) [6].

**Table 1:** Therapeutic properties of *Rauvolfia serpentina* extracts reported for various diseases. Disease details include simple name of the disease and its broad classification. Citations for disease associations are provided in Additional file 2

S. No.	Disease name	Disease type	S. No.	Disease name	Disease type
1	Cancer	Immunological	15	Pneumonia	Pathological
2	Leukemia	Immunological	16	Asthma	Immunological
3	AIDS	Immunological	17	Rheumatism	Immunological
4	Diabetes mellitus	Digestive	18	Anasarca	Epidermal
5	Hypolipidemia	Digestive	19	Helminthiasis	Pathological: Parasitic
6	Alzheimer’s disease	Neurological	20	Cholera	Pathological: Bacterial
7	Schizophrenia	Neurological	21	Cardiac arrhythmia	Circulatory
8	Skin cancer	Immunological	22	Diarrhea	Pathological
9	Prostate cancer	Immunological	23	Tachycardia	Circulatory
10	Hypertension	Circulatory	24	Supraventricular tachysystole	Circulatory
11	Fever	Infectious	25	Thyrotoxicosis	Immunological
12	Insect bite	Infectious	26	Allergy	Immunological
13	Dysentery	Digestive	27	Meningitis	Pathological
14	Malaria	Pathological: Parasitic	28	Encephalitic psychosis	Pathological

**Fig. 1:**
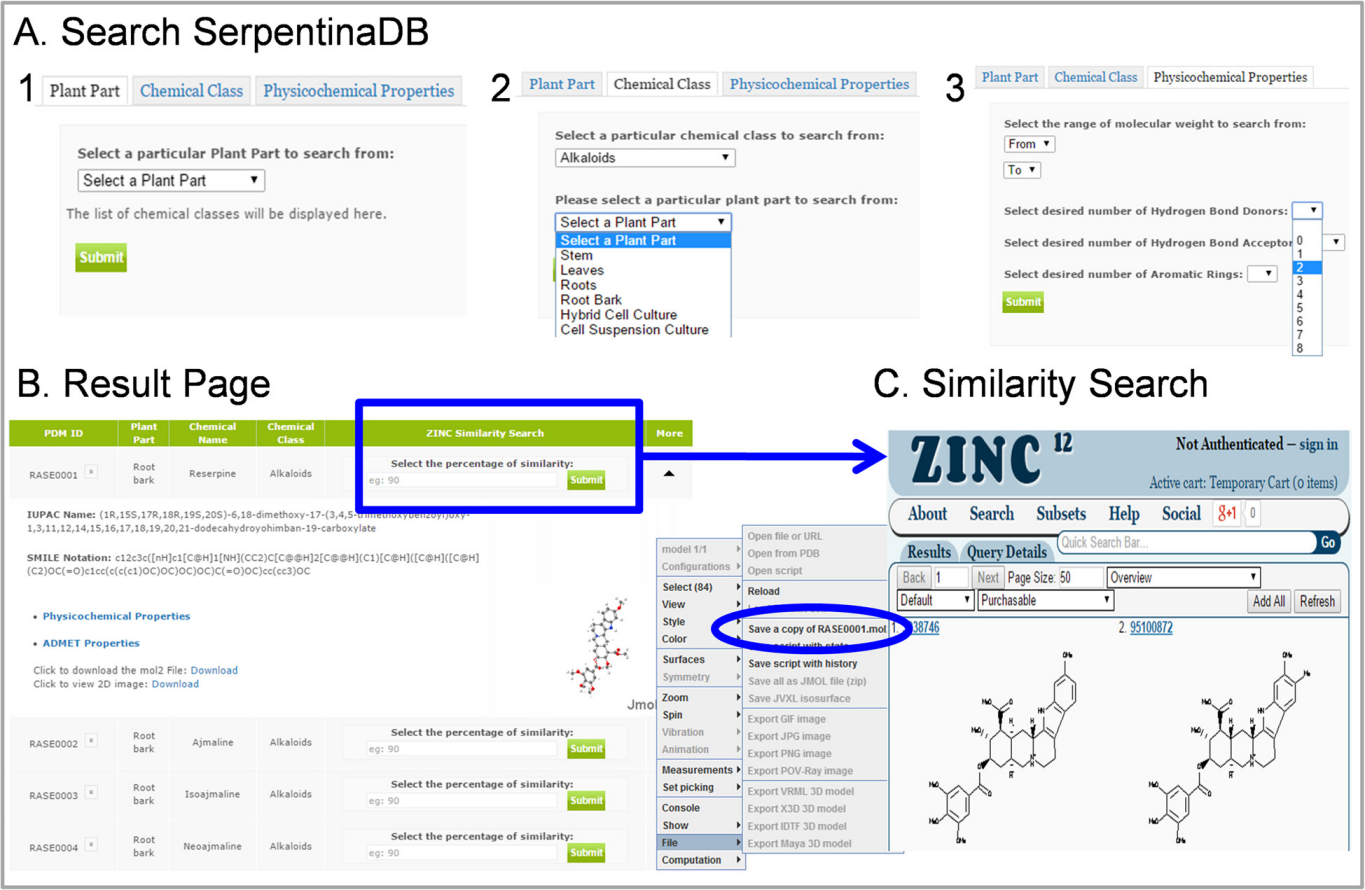
Features of SerpentinaDB interface. (**a**) Demonstration of accessible search options: (1) Plant part, (2) Chemical class as well as plant part, and (3) Physicochemical properties based search of PDMs. (**b**) Result of input query with list of associated PDMs: this page further provides information of IUPAC, SMILES, physicochemical properties, and 3D visualization with associated references. User can download mol2 file and 2D structure of PDM for given query. (**c**) Similarity search of selected PDM against ZINC is also available at user-defined percentage

With the aim of providing a comprehensive resource for rational prospection of *R. serpentina* PDMs towards drug discovery, we compiled an extensive, structured database of its molecules. After a thorough literature survey, details of PDMs were manually compiled and curated. We present a database, SerpentinaDB (Fig. 1), which is structured to include plant part source, chemical name, chemical class, IUPAC (International Union of Pure and Applied Chemistry) names, SMILES (Simplified Molecular-Input Line-Entry System) notations, and 3D (3-Dimensional) structures for 147 PDMs with all associated references (Additional file 2). These 3D structures are present in the form of mol2 file format that is amenable for conversion into other file formats that are accepted by various drug discovery softwares. It also provides several physicochemical descriptors of these PDMs which are indicators of their drug-like properties. Hence access to repertoire of PDMs like SerpentinaDB can be of considerable advantage to academia as well as industry.

## Construction and content

### Data collation and assembly

**Fig. 2:**
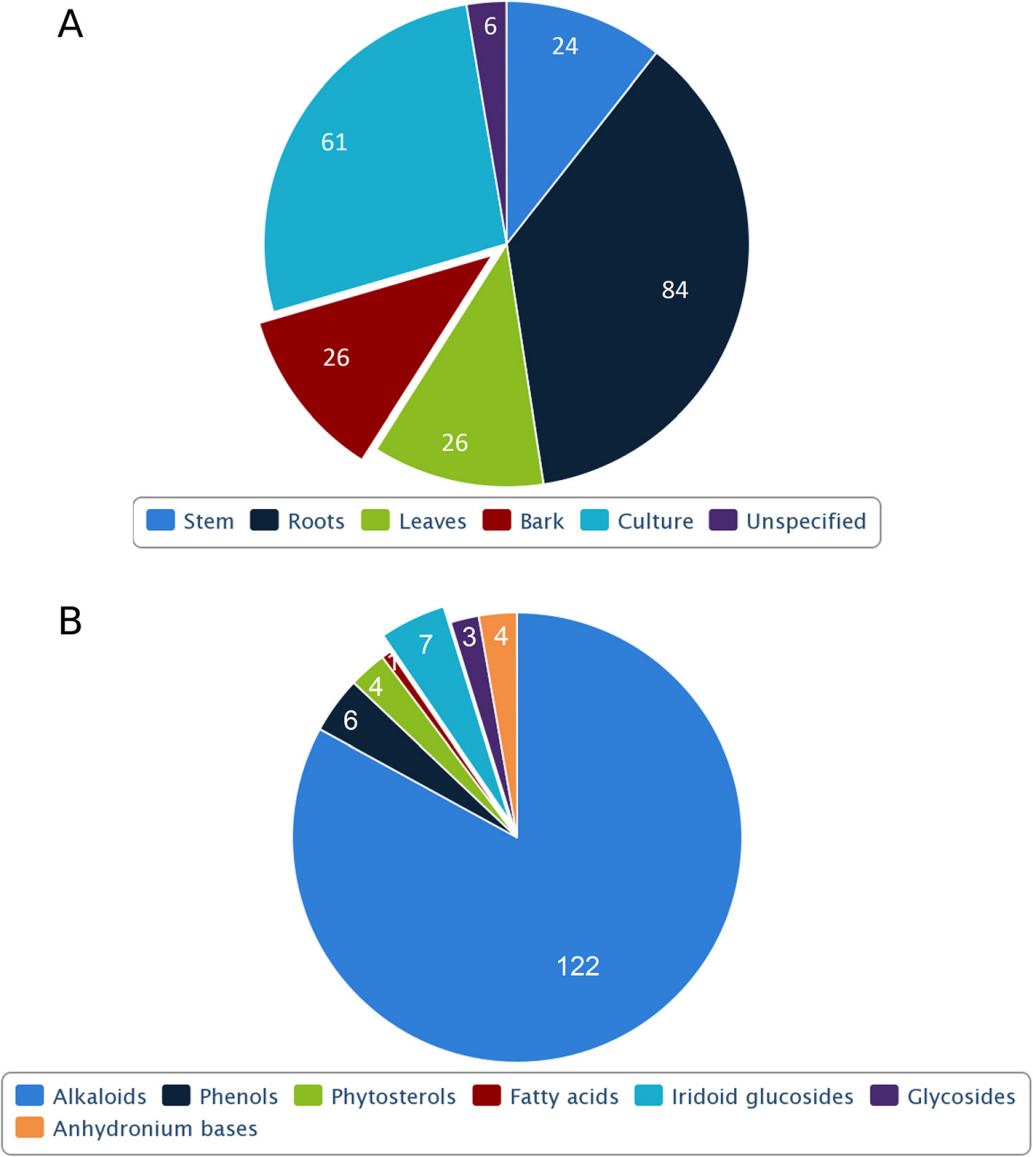
Distribution of *R. serpentina* plant-derived molecules. (**a**) Across various plant parts and (**b**) chemical classes

**Fig. 3:**
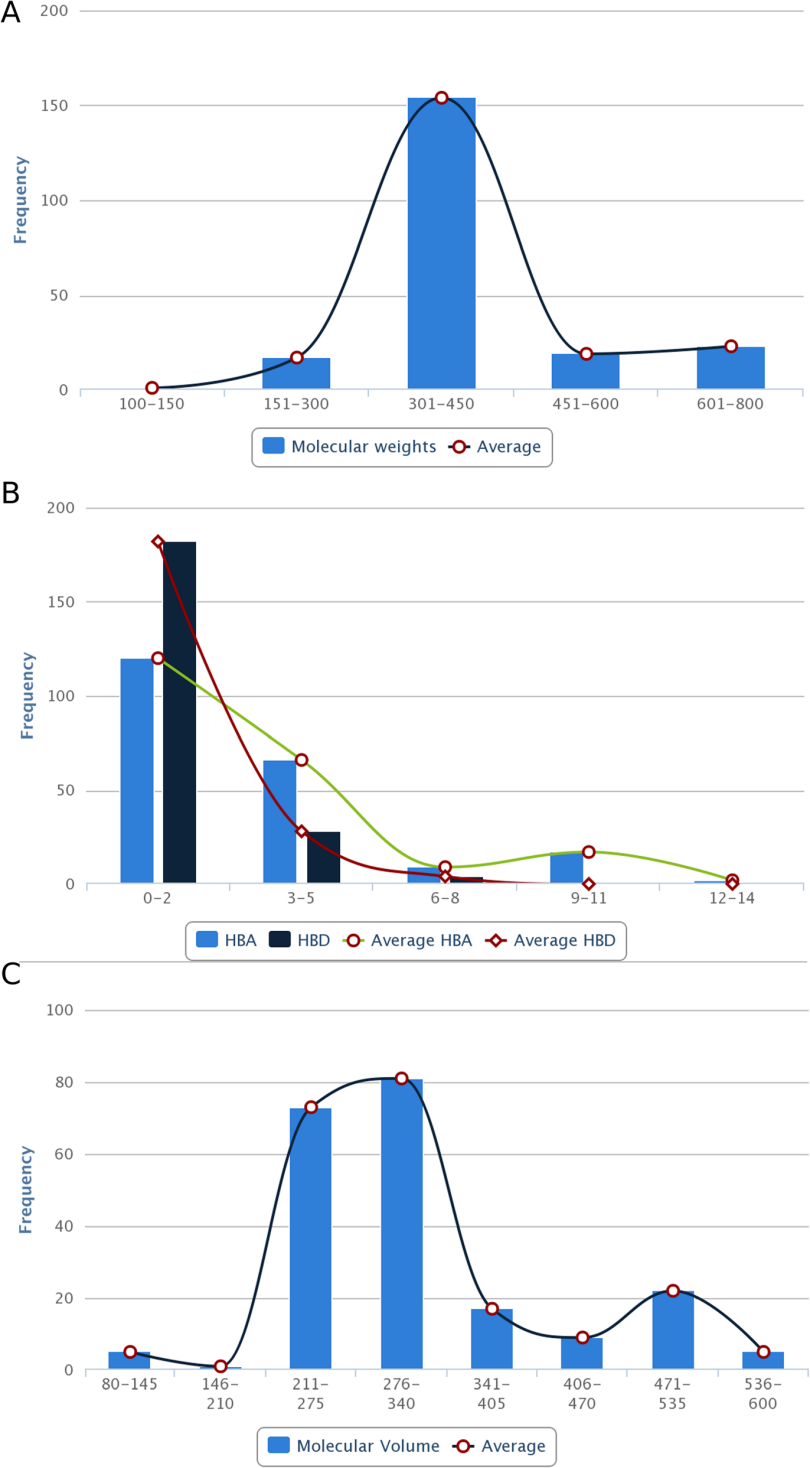
Distribution of molecular descriptors of *R. serpentina* plant-derived molecules. (**a**) Molecular weight. (**b**) Hydrogen bond acceptor and Hydrogen bond donor. (**c**) Molecular volume

In order to build an extensive repository of PDMs from *R. serpentina*, data were compiled from literature and web resources. All resources were manually curated to extract PDMs data and their additional details including plant part, chemical name, chemical class, and IUPAC name. To address degeneracy in the name of the plant, PubMed (http://www.ncbi.nlm.nih.gov/pubmed) was searched with two variants of spelling (‘*Rauvolfia serpentina*’ and ‘*Rauwolfia serpentina*’) to obtain relevant information. A total of 31 research articles, 3 books, 2 PhD dissertations[13, 14], and 3 web resources involving natural compounds research were utilized to compile an extensive list of PDMs. Books and web resources used for curation of dataincluded following sources: ‘The Alkaloids’ [15], ‘The Alkaloids: Chemistry and Physiology’ [16], ‘The Alkaloids: Chemistry and Physiology’ [17], A database on antidiabetic plants [18], Global Information Hub On Integrated Medicine [19], and India Herbs [20]. To authenticate the chemical details obtained, molecules were also ascertained from the Dictionary of Natural Products (DNP) [21], PubChem (https://pubchem.ncbi.nlm.nih.gov/) [22], ChemSpider (http://www.chemspider.com/) [23], and ChEMBL (https://www.ebi.ac.uk/chembl/) [24]. 3D chemical structures of molecules were drawn and edited using MarvinSketch*v*5.10.0 software (https://www.chemaxon.com), and further saved into mol2 file format. These files were subjected to energy minimization with Merck Molecular Force Field (MMFF94) using OpenBabel *v*2.3.1 software [25]. Mol2 format is desirable due to ease of conversion into other molecular formats accepted by drug discovery softwares. To remove redundant entries, data from all resources were merged, and an extensive library of PDMs was compiled. A total of 147 molecules, reported to be extracted from various plant parts, were present in final dataset (Fig. 2a) and broadly classified into different chemical classes (Fig. 2b). Representative PDM of each of seven chemical classes is provided in the supplementary file (Additional file 3). A separate entry was created for molecules that were obtained from more than one plant part leading to 227 such individual entries. Of all these PDMs, mol2 files for 5 of them could not be obtained due to unavailability of both structure as well as IUPAC. Physicochemical properties of the PDMs, such energy, atomic contribution to the partition coefficient (AlogP), distribution coefficient (logD), molecular formula, molecular mass, molecular solubility, and molecular weight (MW), acid dissolution constant (pKa), number of aromatic bonds, number of aromatic rings, and radius of gyration, hydrogen bond acceptor (HBA) count, hydrogen bond donor (HBD) count, number of H acceptor, number of H donor, number of H acceptor (Lipinski), number of H donor (Lipinski), number of H bonds, solvent accessible surface area (SASA), and surface area as well as ADMET (ADMET Solubility, ADMET Solubility Level, ADMET BBB, and ADMET BBB Level) properties, were obtained using molecular property finder tool under small molecules category from the Discovery Studio *v*4.0 (Accelrys, San Diego, USA). Figure 3 illustrates the statistics of various physicochemical properties, such as MW (Fig. 3a), HBA as well as HBD (Fig. 3b), and molecular volume (MV) (Fig. 3c).

**Fig. 4:**
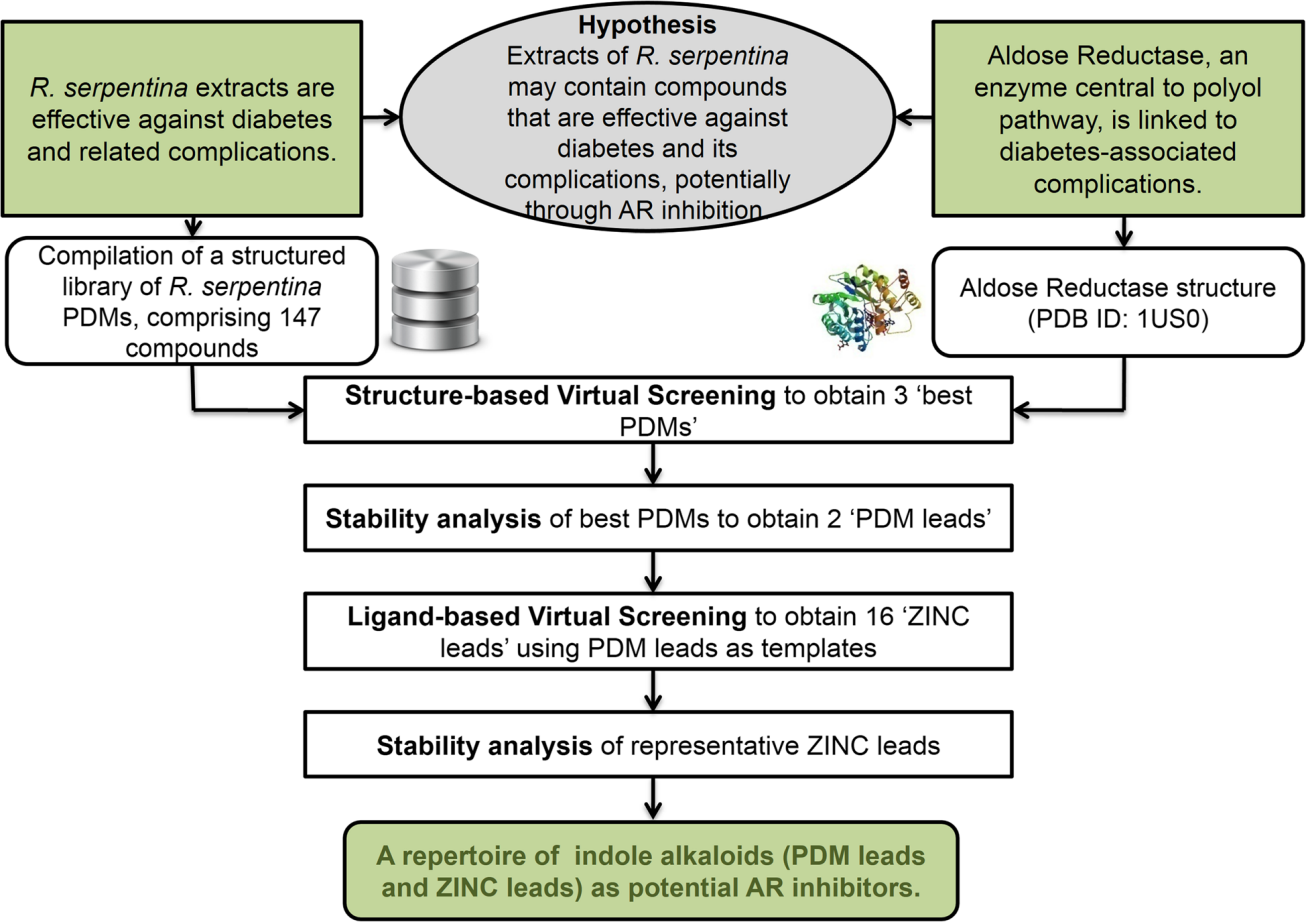
Strategy implemented towards prospecting for novel ARIs from *R. serpentina. R. serpentina* extracts are reported to be effective against diabetes and its complications. Aldose reductase controls the rate-limiting step of polyol pathway, and its inhibition is known to prevent complications of diabetes. Founded in these empirical facts, we proposed a hypothesis connecting effectiveness of molecular constituents of plant extracts to a regulatory mechanism central to the disorder [6]. Towards our aim of prospecting for novel ARIs, we compiled a structured library of *R. serpentina* PDMs, and screened them to obtain ‘best PDMs’ (3). The best PDMs were refined to obtain two ‘PDM leads’ on the basis of their structural stability. Further, 16 more ‘ZINC leads’ were identified by screening structural analogs of these plant-derived leads, and representative analogs were assessed for their structural stability. With this prospection study we presented a repertoire of plant-derived indole alkaloids, and their analogs, as potential AR inhibitors. This study demonstrated the relevance of SerpentinaDB as a structured repertoire of molecules from *R. serpentina* towards hypothesis driven exploration

### Data architecture and Web interface

SerpentinaDB is hosted in a Server at the Indian Institute of Technology Jodhpur on a Dell Power Edge R910 server running a Linux operating system (Red Hat version 5.5). A total of seven data tables were created to house compiled data. SerpentinaDB implements MySQL, an object-relational database management system (RDBMS) for its backend performance. Web browser interface was created using HTML, CSS, Ajax, JavaScript, and jQuery, which connects MySQL terminal using several PHP scripts. A JMol visualizer (http://www.jmol.org/) and ZINC database (http://zinc.docking.org/) has been embedded in Graphical User Interface (GUI) to provide a 3D visualization and percentage similarity search against ZINC, respectively, for all PDMs. The GUI is designed to be user friendly for data query and extraction, and has been tested in all major browsers (Chrome, Firefox, Safari, and Internet Explorer) and OS platforms.

### Data access

SerpentinaDB can be explored for PDMs in a number of ways through querying the database with a simple text search tool that provides various options for searching. There are three search sections available to the user with several constraints in each. Search can be performed with (i) plant part, (ii) chemical class, and (iii) physicochemical properties (Fig. 1a). Physicochemical properties search option has advanced search query options for user to select PDMs in a particular range based on MW, number of HBA, number of HBD, and number of aromatic rings. The result for given query is presented in the same page (Fig. 1b) along with information such as PDM ID, plant part, chemical name, chemical class, IUPAC names, SMILES notations, and 3D structure of PDM with associated references. Clicking the drop down arrow provides details of physicochemical and ADMET properties. Two separate links to download mol2 file and 2-Dimensional structure of PDM for given query has been provided. Also, a JMol visualizer (http://www.jmol.org/) has been embedded in GUI to provide a 3D visualization of PDM which can be further downloaded to mol2 file.

Also, each PDM from result page can be searched, to explore the neighborhood of chemical space, against ZINC database to identify analogs of natural molecules at different percentage similarity cut-off (default 90 %). In order to perform this search ZINC database, a curated collection of commercially available chemical compounds [26], is hyperlinked to result page for each PDM separately to return their structural analogs. During this similarity search natural molecules are used as scaffolds to search for similar mimetics which may have equivalent biological properties. Thus, SerpentinaDB serves as a portal to facilitate the use of natural chemical diversity for drug discovery through prospection of direct novel leads and their analogs.

### Utility and discussion

SerpentinaDB provides comprehensive information of *R. serpentina* PDMs as a structured and integrated library. This database was developed to facilitate prospection of therapeutic molecules from this medicinally important plant. Existing repositories of natural compounds, such as NPACT [27], SuperNatural [28], Herb Ingredients’ Targets [29], and CamMedNP [30], focus on different utilitarian aspects of PDM libraries. While some of these databases emphasize on a specific disease or target-compound interactions, others cover plants of specific geography. SerpentinDB contains natural molecules of *R. serpentina* which is an important Himalayan medicinal plant reported for various pharmacological properties. While the reported efficacy of *R. serpentina* extract against hypertension has been explored very well to identify specific therapeutic PDMs, its potential against a host of other disorders (Table 1) is hitherto not pinned down to specific molecules. This database can facilitate prospection of novel leads for these disorders from the repertoire of natural molecules.

Natural molecules have been recognized to provide specific scaffolds that make them comparable to trade drugs and their potential utilization in combinatorial chemistry [4]. The MW distribution of PDMs present in SerpentinaDB has been found to follow Gaussian distribution and peaked in the range of 300-450 Da (Fig. 3a) which is similar to drug-like molecules of previously reported libraries of natural products [31]. Significant number of PDMs have HBA in the range of 3–5 with a sharp decline thereafter, as desired from drug-like molecules (Fig. 3b). Similarly, HBDs of PDMs have a peak at 2 with a sharp drop for higher values, as desired (Fig. 3b). SerpentinaDB PDMs have maximum density in the ‘Lipinski region of interest’ reflecting their drug-like properties and hence their utility in prospection of novel leads. The relevance of SerpentinaDB in drug discovery has been demonstrated with the virtual screening protocol, molecular dynamics, and ZINC similarity search for potential inhibitors of aldose reductase, a target for complications of diabetes [6]. This hypothesis driven prospection study yielded two indole alkaloids as well as their structural analogs as potential AR inhibitors (Fig. 4) [6]. This protocol serves as a demonstration of utility of SerpentinaDB for rational search of therapeutic molecules and highlights its relevance [32, 33]. Future extensions of SerpentinaDB may include 3D structure similarity search and disease associations for each PDM.

## Conclusions

SerpentinaDB is an exhaustive resource of *R. serpentina* molecules facilitating prospection for therapeutic molecules from a medicinally important source of natural products. Pharmacoinformatics pipeline involving virtual screening to perform docking of molecules against disease specific target to identify inhibitors. Hence, compilation of such datasets is essential step towards *in-silico* drug discovery that hastens the process of prospection of novel leads from natural repertoire with drug-like properties in terms of their biological behavior and toxicity.

### Availability and requirements

SerpentinaDB is available at http://home.iitj.ac.in/~bagler/webservers/SerpentinaDB/. Browsers need to be installed with latest JAVA plugins. For more support please consult the FAQs section of SerpentinaDB.

## Additional files

Additional file 1:
**Alodse reductase inhibitors (‘2 PDM leads’ and their ‘16 structure analogs’) obtained using pharmacoinformatics pipeline in the study performed by Pathania et al., 2013 [** 6 **]**. Additional file 2:
**Details of therapeutic properties of phytochemicals from** ***R. serpentina*** **against various disorders along with associated references.**
Additional file 3:
**Representative PDMs from each of seven chemical classes of** ***R. serpentina*** **.**

## Abbreviations

PDMs: Plant-derived molecules
WHO: World Health Organization
MD: Molecular dynamics
3D: 3Dimensional
1D: 1Dimensional
2D: 2Dimensional
IUPAC: International Union of Pure and Applied Chemistry
SMILES: Simplified molecular-input line-entry system
DNP: Dictionary of Natural Products
MMFF94: Merck Molecular Force Field
AlogP: Partition coefficient
LogD: Distribution coefficient
MW: Molecular weight
PKa: Acid dissolution constant
HBA: Hydrogen bond acceptor
HBD: Hydrogen bond donor
SASA: Solvent accessible surface area
MV: Molecular volume
ADMET: Absorption, distribution, metabolism, excretion, and toxicology
RDBMS: Relational database management system
GUI: Graphical User Interface
OS: Operating System
