# Supplementary material for: SerpentinaDB: a database of plant-derived molecules of Rauvolfia serpentina
Source: BMC Complement Altern Med. 2015 Aug 4;15:262. doi: 10.1186/s12906-015-0683-7 (PMC4523024; doi:10.1186/s12906-015-0683-7)
Supplement: Additional file 1: — Alodse reductase inhibitors (‘2 PDM leads’ and their ‘16 structure analogs’) obtained using pharmacoinformatics pipeline in the study performed by Pathania et al., 2013 [ 6 ]. [file 12906_2015_683_MOESM1_ESM.docx]

**SUPPLEMENTARY INFORMATION**

Title: SerpentinaDB: a database of plant-derived molecules of *Rauvolfia serpentina*

**Shivalika Pathania^1^, Sai Mukund Ramakrishnan^2^, Vinay Randhawa^1,3^ and Ganesh Bagler^1,2,3*^**

^1^Biotechnology Division, CSIR-Institute of Himalayan Bioresource Technology, Council of Scientific and Industrial Research, Palampur, Himachal Pradesh, India

^2^Centre for Biologically Inspired Systems Science, Indian Institute of Technology Jodhpur, India

^3^Academy of Scientific & Innovative Research (AcSIR), New Delhi, India

*Author for correspondence: Phone number: +91-7793820447; fax: +91-291-2449064; email: bagler@iitj.ac.in, ganesh.bagler@gmail.com

**Additional file 1:** Two PDMs of Rauvolfia serpentina obtained as aldose reductase inhibitors along with their sixteen analogs by screening against ZINC database from Pathania *et* al 2013.

| **PDM ID** | **IUPAC** | **STRUCTURE** | |
| --- | --- | --- | --- |
| **PDMs obtained from *R. serpentina* as leads** | | | |
| **RASE0048** | Benzyl 3-(1H-indol-3-yl)propanoate | 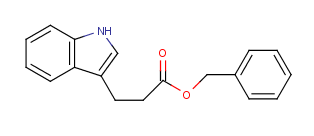 | |
| **RASE0049** | Cyclohexyl 3-(1H-indol-3-yl)propanoate | 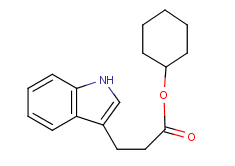 | |
| **ZINC Similarity Search** | | | |
| **ZINC_ID** | **IUPAC** | **STRUCTURE** | |
| **Leads obtained from the analogs of indobine (RASE0048)** | | | |
| ZINC08650120 | Benzyl 3-(1H-indol-3-yl)propanoate | 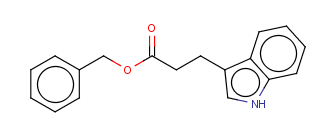 | |
| ZINC04286938 | (3-methylphenyl)methyl 3-(1H-indol-3-yl)propanoate | 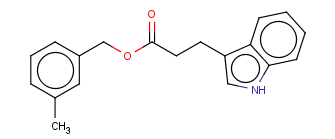 | |
| ZINC08650118 | (4-cyanophenyl)methyl 3-(1H-indol-3-yl)propanoate | 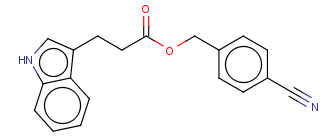 | |
| ZINC04286771 | (4-fluorophenyl)methyl 3-(1H-indol-3-yl)propanoate | 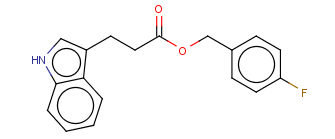 | |
| ZINC08650121 | (4-bromophenyl)methyl 3-(1H-indol-3-yl)propanoate | 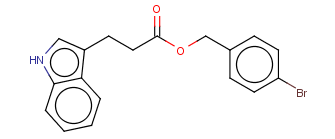 | |
| ZINC04286761 | (4-chlorophenyl)methyl 3-(1H-indol-3-yl)propanoate | 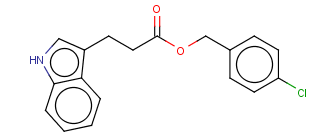 | |
| ZINC03457470 | (2-fluorophenyl)methyl 3-(1H-indol-3-yl)propanoate | 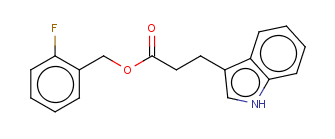 | |
| ZINC04286496 | (3-cyanophenyl)methyl 3-(1H-indol-3-yl)propanoate | 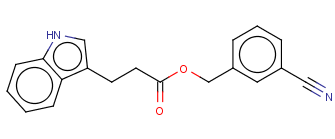 | |
| ZINC03273473 | (3-chlorophenyl)methyl 3-(1H-indol-3-yl)propanoate | 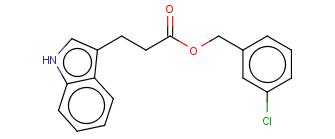 | |
| ZINC08649692 | (3-fluorophenyl)methyl 3-(1H-indol-3-yl)propanoate | 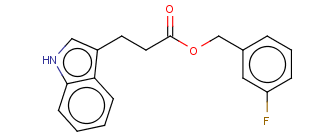 | |
| **Leads obtained from the analogs of indobinine (RASE0049)** | | | |
| ZINC14517206 | Cyclohexyl 3-(1H-indol-3-yl)propanoate | | 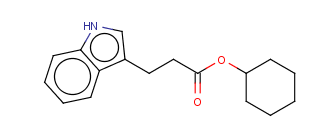 |
| ZINC66777292 | (1R,3S)-3-methoxycyclohexyl 3-(1H-indol-3-yl)propanoate | | 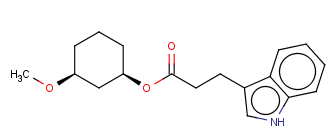 |
| ZINC49016166 | Cyclopentyl 3-(1H-indol-3-yl)propanoate | | 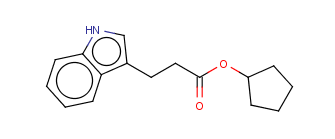 |
| ZINC04286775 | (1S)-2-oxocyclohexyl 3-(1H-indol-3-yl)propanoate | | 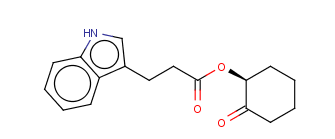 |
| ZINC04286774 | (1R)-2-oxocyclohexyl 3-(1H-indol-3-yl)propanoate | | 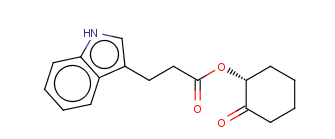 |
| ZINC57967803 | (2S)-oxan-2-ylmethyl 3-(1H-indol-3-yl)propanoate | | 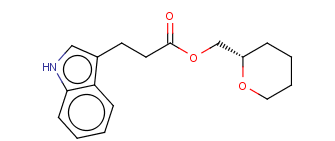 |
