# Supplementary material for: SerpentinaDB: a database of plant-derived molecules of Rauvolfia serpentina
Source: BMC Complement Altern Med. 2015 Aug 4;15:262. doi: 10.1186/s12906-015-0683-7 (PMC4523024; doi:10.1186/s12906-015-0683-7)
Supplement: Additional file 2: — Details of therapeutic properties of phytochemicals from R. serpentina against various disorders along with associated references. [file 12906_2015_683_MOESM2_ESM.docx]

**SUPPLEMENTARY INFORMATION**

Title: SerpentinaDB: a database of plant-derived molecules of *Rauvolfiaserpentina*

**Shivalika Pathania^1^, Sai Mukund Ramakrishnan^2^, Vinay Randhawa^1,3^ and Ganesh Bagler^1,2,3*^**

^1^Biotechnology Division, CSIR-Institute of Himalayan Bioresource Technology, Council of Scientific and Industrial Research, Palampur, Himachal Pradesh, India

^2^Centre for Biologically Inspired Systems Science, Indian Institute of Technology Jodhpur, India

^3^Academy of Scientific & Innovative Research (AcSIR), New Delhi, India

*Author for correspondence: Phone number: +91-7793820447; fax: +91-291-2449064; email: bagler@iitj.ac.in, ganesh.bagler@gmail.com

**Additional file 2:** Therapeutic properties of phytochemicals of *Rauvolfiaserpentina* in various diseases with associated references. Disease details include simple name of the disease and its broad classification.

| **S. No.** | **Disease name** | **Disease type** | **References** |
| --- | --- | --- | --- |
| 1 | Cancer | Immunological | [1–4] |
| 2 | Leukemia | Immunological | [5] |
| 3 | AIDS | Immunological | [6, 7] |
| 4 | Diabetes mellitus | Digestive | [8–11] |
| 5 | Hypolipidemia | Digestive | [12, 8] |
| 6 | Alzheimer’s disease | Neurological | [13] |
| 7 | Schizophrenia | Neurological | [7, 14–16] |
| 8 | Skin cancer | Immunological | [3, 7] |
| 9 | Prostate cancer | Immunological | [2, 17] |
| 10 | Hypertension | Circulatory | [7, 18–22] |
| 11 | Fever | Infectious | [23] |
| 12 | Insect bite | Infectious | [23] |
| 13 | Dysentery | Digestive | [23] |
| 14 | Malaria | Pathological: Parasitic | [24, 25] |
| 15 | Pneumonia | Pathological | [26] |
| 16 | Asthma | Immunological | [27] |
| 17 | Rheumatism | Immunological | [7, 28] |
| 18 | Anasarca | Epidermal | [28] |
| 19 | Helminthiasis | Pathological: Parasitic | [29, 30] |
| 20 | Cholera | Pathological: Bacterial | [29, 30] |
| 21 | Cardiac arrhythmia | Circulatory | [31–33] |
| 22 | Diarrhea | Pathological | [34] |
| 23 | Tachycardia | Circulatory | [35] |
| 24 | Supraventricular tachysystole | Circulatory | [36] |
| 25 | Thyrotoxicosis | Immunological | [35] |
| 26 | Allergy | Immunological | [37, 38] |
| 27 | Meningitis | Pathological | [39] |
| 28 | Encephalitic psychosis | Pathological | [39] |

**References**

1. Beljanski M, Beljanski MS: **Selective inhibition of in vitro synthesis of cancer DNA by alkaloids of beta-carboline class**. *Exp Cell Biol* 1982, **50**:79–87.

2. Beljanski M, Beljanski MS: **Three alkaloids as selective destroyers of cancer cells in mice. Synergy with classic anticancer drugs**. *Oncology* 1986, **43**:198–203.

3. Harisaranraj R, Suresh K, Babu SS, Achudhan VV: **Phytochemical based strategies for pathogen control and Antioxidant Capacities of Rauwolfia serpentina Extracts**. *Recent Res Sci Technol* 2009, **1**:67–78.

4. Arts IC, Hollman PC: **Polyphenols and disease risk in epidemiologic studies**. *Am Soc Clin Nutr* 2005, **81**(1 Suppl):317S–325S.

5. Itoh A, Kumashiro T, Yamaguchi M, Nagakura N, Mizushina Y, Nishi T, Tanahashi T: **Indole alkaloids and other constituents of Rauwolfia serpentina**. *J Nat Prod* 2005, **68**:848–852.

6. Rahmatullah M, Jahan R, Azad AK, Seraj S, Rahman MM, Chowdhury AR, Begum R, Nasrin D, Khatun Z, Mohammad, Hossain S, Khatun MA, Miajee ZUME: **Medicinal plants used by folk medicinal practitioners in three villages of Natore and Rajshahi districts, Bangladesh**. *Am J Sustain Agric* 2010, **4**:211–218.

7. Dey A, De J: **Ethnobotanical aspects of Rauvolfia serpentina (L). Benth. ex Kurz. in India, Nepal and Bangladesh**. *J Med Plants Res* 2011, **5**:144–150.

8. Qureshi S, Nawaz A, Udani S, Azmi B: **Hypoglyceamic and Hypolipidemic Activities of Rauwolfia serpentina in Alloxan-Induced Diabetic Rats**. *Int J Pharmacol* 2009, **5**:323–326.

9. Azmi M, Qureshi A: **Methanolic root extract of Rauwolfia serpentina improves the glucose tolerance in wister mice**. *J Food Drug Anal* 2012, **20**:484–488.

10. Ganugapati J, Baldwa A, Lalani S: **Docking Studies of Rauwolfia Serpentina Alkaloids as Insulin Receptor Activators**. *Int J Comput Appl* 2012, **43**:32–37.

11. Pathania S, Randhawa V, Bagler G: **Prospecting for novel plant-derived molecules of Rauvolfia serpentina as inhibitors of Aldose Reductase, a potent drug target for diabetes and its complications**. *PLoS One* 2013, **8**:e61327.

12. Azmi MB, Qureshi SA: **Methanolic Root Extract of Rauwolfia serpentina Benth Improves the Glycemic, Antiatherogenic, and Cardioprotective Indices in Alloxan-Induced Diabetic Mice**. *Adv Pharmacol Sci* 2012, **2012**:1–11.

13. Mathew M, Subramanian S: **In vitro screening for anti-cholinesterase and antioxidant activity of methanolic extracts of ayurvedic medicinal plants used for cognitive disorders**. *PLoS One* 2014, **9**:e86804.

14. Preenon B, Ajit K, Vinobha CS: **Establishing an in-silico ayurvedic medication towards treatment of Schizophrenia**. *Int J Syst Biol* 2009, **1**:46–50.

15. Pletscher A, Shore PA, Brodie BB: **Serotonin as a mediator of reserpine action in brain**. *J Pharmacol Exp Ther* 1956, **116**:84–89.

16. Charveron M, Assié M-B, Stenger A, Briley M: **Benzodiazepine agonist-type activity of raubasine, a rauwolfia serpentina alkaloid**. *Eur J Pharmacol* 1984, **106**:313–317.

17. Bemis DL, Capodice JL, Gorroochurn P, Katz AE, Buttyan R: **Anti-prostate cancer activity of a β-carboline alkaloid enriched extract from Rauwolfia vomitoria**. *Int J Oncol* 2006, **29**:1065–1073.

18. Nammi S, Boini KM, Koppula S, Sreemantula S: **Reserpine-induced central effects: pharmacological evidence for the lack of central effects of reserpine methiodide**. *Can J Physiol Pharmacol* 2005, **83**:509–515.

19. Kumaria R, Rathib B, Ranic A, Bhatnagar S: **Rauvolfia serpentina L. Benth. ex Kurz.: phytochemical, pharmacological and therapeutic aspects**. *Int J Pharm Sci Rev Res* 2013, **23**:348–355.

20. Khare CP: *Indian Medicinal Plants: An Illustrated Dictionary*. Springer; 2007:900.

21. Bhatara VS, Sharma JN, Gupta S, Gupta YK: **Images in psychiatry. Rauwolfia serpentina: the first herbal antipsychotic**. *Am J Psychiatry* 1997, **154**:894.

22. Vakil R: **Rauwolfia Serpentina in the Treatment of High Blood Pressure; A Review of the Literature**. *Circulation* 1955, **12**:220–229.

23. Nayak S, Behera SK, Misra MK: **Ethno-medico-botanical survey of Kalahandi district of Orissa**. *Indian J Tradit Knowl* 2004, **3**:72–79.

24. Anisuzzaman M, Rahman a. HMM, Harun-Or-Rashid M, Naderuzzaman a. TM, Islam a. KMR: **An ethnobotanical study of Madhupur, Tangail**. *J Appl Sci Res* 2007, **3**:519–530.

25. Dassonneville L, Bonjean K, De Pauw-Gillet MC, Colson P, Houssier C, Quetin-Leclercq J, Angenot L, Bailly C: **Stimulation of topoisomerase II-mediated DNA cleavage by three DNA-intercalating plant alkaloids: cryptolepine, matadine, and serpentine**. *Biochemistry* 1999, **38**:7719–7726.

26. Rai SK: **Medicinal Plants used by Meche People of Jhapa District, Eastern Nepal**. *Our Nat* 2004, **2**:27–32.

27. De Britto J, Mahesh R: **Exploration of Kani Tribal Botanical Knowledge in Agasthiayamalai Biosphere Reserve - South India**. *Ethnobotanical Leaflets* 2007:258–265.

28. Behera SK, Panda A, Behera SK, Misra MK: **Medicinal plants used by the Kandhas of Kandhamal district of Orissa**. *Indian J Tradit Knowl* 2006, **5**:519–528.

29. Italie V: **On the Use of Rauwolfia serpentina in High Blood Pressure**. *J Indian Med Assoc* 1942, **11**:262–265.

30. **GLOBinMeD-Global Information Hub On Integrated Medicine.** [http://www.globinmed.com/]

31. Bazika V: **Ajmaline, an alkaloid of Rauwolfia serpentina, in the treatment of cardiac arrhythmias**. *Vnitr Lek* 1969, **15**:662–670.

32. Köppel C, Wagemann A, Martens F: **Pharmacokinetics and antiarrhythmic efficacy of intravenous ajmaline in ventricular arrhythmia of acute onset**. *Eur J Drug Metab Pharmacokinet* 1989, **14**:161–167.

33. Kleinsorge H, Wittig HH: **The therapy of cardiac and circulatory diseases with the alkaloids of Rauwolfia serpentina**. *Rev Bras Med* 1962, **19**:1–7.

34. Ezeigbo II, Ezeja MI, Madubuike KG, Ifenkwe DC, Ukweni IA, Udeh NE, Akomas SC: **Antidiarrhoeal activity of leaf methanolic extract of Rauwolfia serpentina**. *Asian Pac J Trop Biomed* 2012, **2**:430–432.

35. Jerie P: **Milestones of cardiovascular therapy IV. Reserpine**. *Ceskoslov Lek Spol* 2007, **146**:573–577.

36. Kel’man IM, Paleev NR: **Prevention of supraventricular tachysystole with anti-arrhythmia agents**. *Kardiologiia* 1977, **17**:42–45.

37. Lelek I, Furedi Szabo M: **Data on the anti-allergic activity of Rauwolfia serpentina alkaloid reserpine**. *Orv Hetil* 1960, **101**:1233–1234.

38. Lelek I, Furedi Szabo M: **On the antiallergic effect of the reserpine alkaloid of Rauwolfia serpentina**. *Allerg Asthma (Leipz)* 1961, **7**:142–144.

39. Abad MB: **Rauwolfia serpentina in meningitic and encephalitic psychosis in children**. *J Philipp Med Assoc* 1959, **35**:429–434.
