# Supplementary material for: SerpentinaDB: a database of plant-derived molecules of Rauvolfia serpentina
Source: BMC Complement Altern Med. 2015 Aug 4;15:262. doi: 10.1186/s12906-015-0683-7 (PMC4523024; doi:10.1186/s12906-015-0683-7)

**SUPPLEMENTARY INFORMATION**

Title: SerpentinaDB: a database of plant-derived molecules of *Rauvolfiaserpentina*

**Shivalika Pathania^1^, Sai Mukund Ramakrishnan^2^, Vinay Randhawa^1,3^ and Ganesh Bagler^1,2,3*^**

^1^Biotechnology Division, CSIR-Institute of Himalayan Bioresource Technology, Council of Scientific and Industrial Research, Palampur, Himachal Pradesh, India

^2^Centre for Biologically Inspired Systems Science, Indian Institute of Technology Jodhpur, India

^3^Academy of Scientific & Innovative Research (AcSIR), New Delhi, India

*Author for correspondence: Phone number: +91-7793820447; fax: +91-291-2449064; email: bagler@iitj.ac.in, ganesh.bagler@gmail.com

**Additional file 3:** Representative PDMs from each of seven chemical classes of *R. serpentina*.


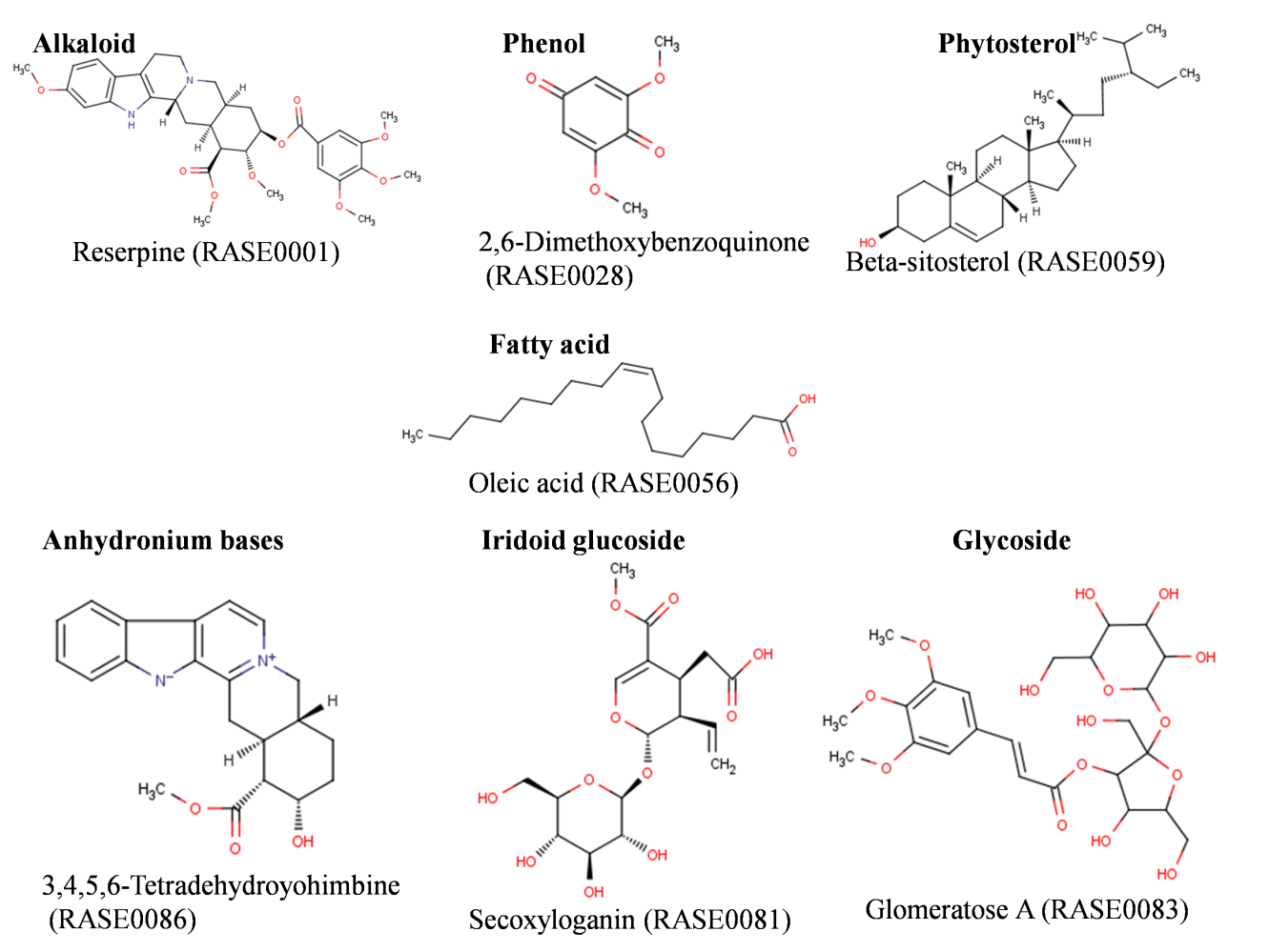

Supplement: Additional file 3: — Representative PDMs from each of seven chemical classes of R. serpentina . [file 12906_2015_683_MOESM3_ESM.docx]
